# Complete mitochondrial genome of *Carijoa riisei* (Duchassaing & Michelotti, 1860) (Octocorallia: Alcyonacea: Stolonifera: Clavulariidae)

**DOI:** 10.1080/23802359.2020.1750998

**Published:** 2020-04-13

**Authors:** Erin E. Easton, David Hicks

**Affiliations:** School of Earth, Environmental and Marine Sciences, University of Texas Rio Grande Valley, Brownsville, TX, USA

**Keywords:** Artificial reef, Cnidaria, Gulf of Mexico, mesophotic

## Abstract

We report the first complete Stolonifera mitochondrial genome. *Carijoa riisei* (Duchassaing & Michelotti, 1860) isolate CLP2_A03 was collected by scuba at 32 m on the USTS *Texas Clipper* (27° 53.7827′N, 93° 36.2702′W). The complete mitogenome has the ancestral octocoral gene order for its 14 protein-coding genes, two rRNA genes, and one tRNA gene. It is 18,714 bp (30.7% A, 15.8% C, 18.8% G, and 34.7% T). Of the Alcyonacea mitogenomes published to date, it is most genetically similar (94% uncorrected) to *Sinularia ceramensis* Verseveldt, 1977 (NC_044122).

*Carijoa* Müller, 1867 consists of three accepted species of which two, *C. riisei* and *C. operculata*, are reported in the Gulf of Mexico. These species are distinguishable by anthocodial spiculation, which forms an operculum in the latter species (Bayer 1961). Although *C. riisei* was described from the Atlantic-Caribbean, it is widely distributed in the Atlantic, Pacific, and Indian Oceans and has a likely origin in the Indo-Pacific, whereas, the Atlantic and Caribbean populations are inferred to be the youngest (Concepcion et al. 2010). Colonies can reach sexual maturity in a few months, can maintain growth rates ∼0.5 cm week^−1^ for several months, undergo vegetative propagation, and are gonochoristic (rarely hermaphroditic) with continuous and asynchronous gamete release (Kahng et al. 2008; Barbosa et al. 2014). These characteristics enable *C. riisei* to proliferate rapidly and to form dense aggregations and may contribute to its colonization of artificial structures (Kahng et al. 2008).

The specimen was collected by scuba at 32 m on the USTS *Texas Clipper* (27° 53.7827′N, 93° 36.2702′W) on 22 August 2017. This vessel was sunk as part of the Texas Parks and Wildlife Department’s Artificial Reef Program on 17 November 2007 (Curley 2011). DNA was extracted with GeneJET Genomic DNA Purification Kit (ThermoFisher Scientific, Waltham, MA) per manufacture’s protocol and submitted to Biopolymers Facility at Harvard Medical School for library preparation (Illumina Nextera XT2) and next-generation sequencing (NextSeq 500). Trimmed reads (Trimmomatic-0.32, Bolger et al. 2014) were assembled de novo with SPAdes (Bankevich et al. 2012) on the University of New Hampshire ron server. Trimmed reads (BBDuk v. 37.25) were mapped to the SPAdes contig to generate a consensus sequence in Geneious Prime 2020.0.5 (https://www.geneious.com). Genes were annotated by manually adjusting *Muricea crassa* Verrill, 1869 (NC029697) annotations mapped to the consensus sequence in Geneious. The *C. riisei* mitogenome was deposited in GenBank (MT161608) and the specimen and sclerite SEM plate (see Supplemental Material) were deposited in the Smithsonian National Museum of National History (USNM1616994). The complete mitogenome was aligned with default MUSCLE (Edgar 2004) parameters in Geneious to 26 representative species for which the complete mitochondrial genomes with the ancestral gene order were available in GenBank. A maximum-likelihood, phylogenetic tree, rooted with the Pennatulacea clade, was constructed with RaxML 8.2.11 (Stamatakis 2014) (Figure 1): 100 bootstrap replicates (rapid bootstrapping with search for best-scoring ML tree), no outgroup, and nucleotide model = GTR CAT I. Extended methods and alignments are available in Supplemental Material.

**Figure 1.**
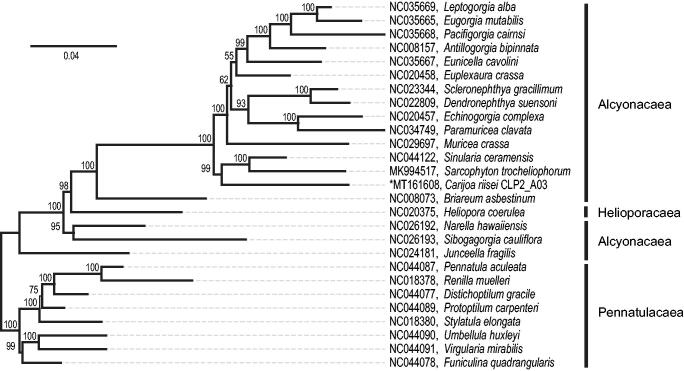
Maximum-likelihood, phylogenetic tree of the complete mitochondrial genomes of *Carijoa riisei* (*this study, GenBank accession number, species name, field sample ID) and 26 representative octocorals (GenBank accession number, species name). In Geneious Prime 20.0.5, complete mitochondrial genomes were aligned with default MUSCLE parameters; the resulting alignment was used to construct the phylogenetic tree with RaxML 8.2.11 plugin with the following changes to the default settings: bootstrap replicates = 100, algorithm = rapid bootstrapping and search for best-scoring ML tree, nucleotide model = GTR CAT I. Bootstrap values >50 are report at the nodes. See Supplemental Material for methods details.

The complete mitogenome is 18,714 bp (30.7% A, 15.8% C, 18.8% G, and 34.7% T), has the ancestral octocoral gene order, and has 14 protein-coding genes, two rRNA genes, and one tRNA gene. This mitogenome report is the first for suborder Stolonifera. *C. riisei* is sister to the two species in Alcyoniidae and ∼94% similar (uncorrected) to *Sinularia ceramensis* Verseveldt, 1977 (Alcyoniina: Alcyoniidae) (Figure 1). As found in previous studies (Figueroa and Baco 2015; Poliseno et al. 2017), phylogenetic reconstructions result in polyphyly of octocoral taxa, including orders, such as Alcyonacaea (Figure 1).

## Supplementary Material

Supplemental Material
