# Supplementary material for: Complete mitochondrial genome of Carijoa riisei (Duchassaing & Michelotti, 1860) (Octocorallia: Alcyonacea: Stolonifera: Clavulariidae)
Source: Mitochondrial DNA B Resour. 2020 Apr 13;5(2):1826–7. doi: 10.1080/23802359.2020.1750998 (PMC7577009; doi:10.1080/23802359.2020.1750998)
Supplement: Supplemental Material [file TMDN_A_1750998_SM4098.docx]

**Supplemental files for the publication**

**Complete mitochondrial genome of *Carijoa* *riisei* (Octocorallia: Alcyonacea: Stolonifera:** **Clavulariidae)**

**Erin E. Easton and David Hicks**

**School of Earth, Environmental, and Marine Sciences, University of Texas Rio Grande Valley, Brownsville, TX, USA**

**Specimen CLP2_A03 deposited to the Smithsonian National Museum of National History: USNM 1616994**

**Complete mitogenome sequence deposited to GenBank: MT161608**

**Correspondence details: UTRGV-SEEMS, 33363 Marine Lab Dr., South Padre Island, TX 78597;** [**erin.easton@utrgv.edu**](mailto:erin.easton@utrgv.edu)

**Methods**

The specimen was collected by scuba at 32 m on the USTS *Texas Clipper* (27° 53.7827’ N, 93° 36.2702’ W) on 22 August 2017. This vessel was sunk as part of the Texas Parks and Wildlife Artificial Reef Program on 17 November 2007 (Curley 2011). The specimen was stored in pure ethanol at -20°C. DNA was extracted from three polyps with GeneJET Genomic DNA Purification Kit (ThermoFisher Scientific Waltham, MA) per manufacture’s protocol. Extracted DNA was submitted to Biopolymers Facility at Harvard Medical School for library preparation (Illumina Nextera XT2) and next-generation sequencing (NextSeq 500). Trimmed reads (Trimmomatic-0.32, Bolger et al. 2014) were assembled de novo by SPAdes 3.13.0 (Bankevich et al. 2012) on the University of New Hampshire Bioinformatics Core facility ron server, which is supported by the New Hampshire-INBRE Program through an Institutional Development Award, P20GM103506, from the National Institute of General Medical Sciences of the NIH. See Trimmomatic and SPAdes scripts below for settings. To identify the mitochondrial genome, we conducted a blastn search of the resulting SPAdes contigs against all reference mitochondrial genomes in GenBank downloaded to the ron server. The identified contig was then imported into Geneious Prime 20.0.05 (<https://www.geneious.com>) and circularized. We used the Find repeats option in Geneious to identify any repeats >15 bp. Consecutive repeats at the junction of the ends of the SPAdes contig were reviewed manually and one repeat, presumed to be an artefact of assembly, was deleted. To confirm this assumption, reads were mapped to this sequence in Geneious as follows. Reads were paired with default settings for Illumina paired ends as implemented in Geneious except Expected Distance = 250. Paired reads were trimmed with BBDuk v. 38.37 as implemented in Geneious. BBDuk default settings were used except the following options were selected (see below for complete commands): Trim adapters (Ilumina Nextera adapters – 71 sequences) and Trim Low Quality (Both Ends, Minimum Quality = 6). BBDuk-trimmed reads were mapped to the edited SPAdes contig in Geneious to generate a consensus sequence with the following settings: Mapper = Geneious, Sensitivity = Medium-Low Sensitivity/Fast, Fine Tuning = None (fast/read mapping), Trim Before Mapping = Do Not Trim, Map multiple best matches = Randomly, Trim paired read overhangs = checked, Allow Gaps = checked (Maximum Per Read = 10%, Maximum Gap Size = 15), Word length = 18, Index word length = 13, Ignore words repeated more than = checked (12 times), Maximum mismatches per read = 20%, Maximum Ambiguity = 4, and Accurately map reads with errors to repeat regions = checked. A consensus sequence was generated with Threshold = 90% and Assign Quality = Total.

Genes were annotated with Live Annotate and Predict in Geneious with *Muricea crassa* Verrill, 1869 (Alcyonacea: Holaxonia: Plexauridae; GenBank accession NC029697) annotations, and annotations were manually adjusted based on alignments with genes extracted from available Octocorallia and Hexacorallia complete mitogenomes, including those with different gene orders. Numerous alternative incomplete and complete stop codons were identified for cox1, so we chose the first candidate that would be present in all Hexacorallia and Octocorallia mitogenomes available but acknowledge the inferred stop codon could be up to 190 bp downstream, where a complete stop codon was identified at position 117-119 of the inferred 12S rRNA gene. The most common incomplete stop codon inferred among Octocorallia mitogenomes that did not have a complete stop codon before the inferred 12S rRNA gene start, is 77 bp downstream of the stop inferred for *C. riisei* and overlaps six bases of the start of the inferred 12S gene in in this study. Likewise, alternative boundaries of the 12S and 16S rRNA genes were inferred based off alignments with Octocorallia and Hexacorallia complete mitogenomes. Inferred gene boundaries were selected by choosing the potential starts and stop positions best conserved for these genes across all the available complete Octocorallia and Hexacorallia mitogenomes available.

The complete mitogenome was aligned with default MUSCLE (Edgar 2004) parameters in Geneious representative species with the inferred ancestral mitogenome gene order for which the complete mitochondrial genomes were available in GenBank. We selected 26 species to ensure coverage of most genera and families represented by the 81 available mitogenome sequence, with the first published reference mitogenome per genus selected instead of the more recently published mitogenomes The mitogenomes used include Pennutalidae species *Funiculina quadrangularis* (Pallas, 1766) (NC_044078), *Distichoptilum gracile* Verrill, 1882 (NC_044077), *Protoptilum carpenteri* Kölliker, 1872 (NC_044089), *Renilla muelleri* Kölliker, 1872 (NC_018378), *Umbellula huxleyi* Kölliker, 1880 (NC_044090), *Pennatula aculeata* Danielssen, 1860 (NC_044087), *Stylatula elongata* Verrill, 1864 (NC_018380), and *Virgularia mirabilis* (Müller, 1776) (NC_044091); Helioporacea species *Heliopora coerulea* (Pallas, 1766) (NC_020375); and Alcyonacaea species *Sarcophyton trocheliophorum* von Marenzeller, 1886 (MK994517), *Sinularia ceramensis* Verseveldt, 1977 (NC_044122), *Dendronephthya suensoni* (Holm, 1895) (NC_022809), *Junceella fragilis* (Ridley, 1884) (NC_024181), *Narella hawaiinensis* Cairns & Bayer, 2007 (NC_026192), *Antillogorgia bipinnata* (Verrill, 1864) (NC_008157), *Eugorgia mutabilis* Breedy, Williams & Guzman, 2013 (NC_035665), *Eunicella cavolini* (Koch, 1887) (NC_035667), *Leptogorgia alba* (Duchassaing & Michelotti, 1864) (NC_035669), *Pacifigorgia cairnsi* Breedy & Guzman, 2003 (NC_035668), *Paramuricea clavata* (Risso, 1826) (NC_034749), *Echinogorgia complexa* Nutting, 1910 (NC_020457), *Euplexaura crassa* Kükenthal, 1908 (NC_020458), *Muricea crassa* Verrill, 1869 (NC_029697), *Briareum asbestinum* (Pallas, 1766) (NC_008073), *Sibogagorgia cauliflora* Herrera, Baco & Sánchez, 2010 (NC_026193). To select the best model of nucleotide evolution, we used PartitionFinder 2.1.1 (Lanfear et al. 2017) with the following settings: branchlengths=unlinked, models = all with --raxml code, model_selection = aicc, search = greedy (Lanfear et al. 2012), and data blocks defined to the common extent of each gene (tRNA, rRNA, and protein-coding genes) among all taxa in the alignment with protein-coding genes split into the three codon positions and each intergenic region (IGR) defined as separate blocks for a total of 61 data blocks. When PartitionFinder was rerun on the alignment with all IGRs excluded, the same best model (GTR+I+G) was selected for all within-gene data blocks. A maximum-likelihood, phylogenetic tree was constructed in RAxML 8.2.11 (Stamatakis 2014) based on the allignment of the complete mitogenomes, including IGRs: 100 bootstrap replicates (rapid bootstrapping with search for best-scoring ML tree), no outgroup, nucleotide model = GTR CAT I. The best tree was then rooted with the Pennatulacae clade.

An ~ 1 cm (length) sample of CLP2_A03 was prepared for examination on the scanning electron microscope (SEM) by removing tissue with a treatment of 8.25% sodium hypochlorite solution. Once all tissue was dissolved, a treatment of 3% hydrogen peroxide was applied to the remaining sclerites. The sclerites were then rinsed with deionized water and treated with 95% ethanol to accelerate drying. Dried sclerites were adhered to an aluminum stub with double-sided carbon tape and coated in a 10 nm layer of Gold/Palladium with a Denton Vacuum Desk V (Moorestown, NJ) sputter coater. The sclerites were then analysed and imaged using a JEOL JSM5600LV SEM (JEOL USA Incorporated,Peabody, USA). Sclerite morphology (see Plate below) is consistent with that described and illustrated in Bayer (1961) for this species collected from the Caribbean.

The *C. riisei* mitogenome was deposited in GenBank (MT161608) and the specimen and sclerite SEM plate were deposited in the Smithsonian National Museum of National History (USNM1616994).


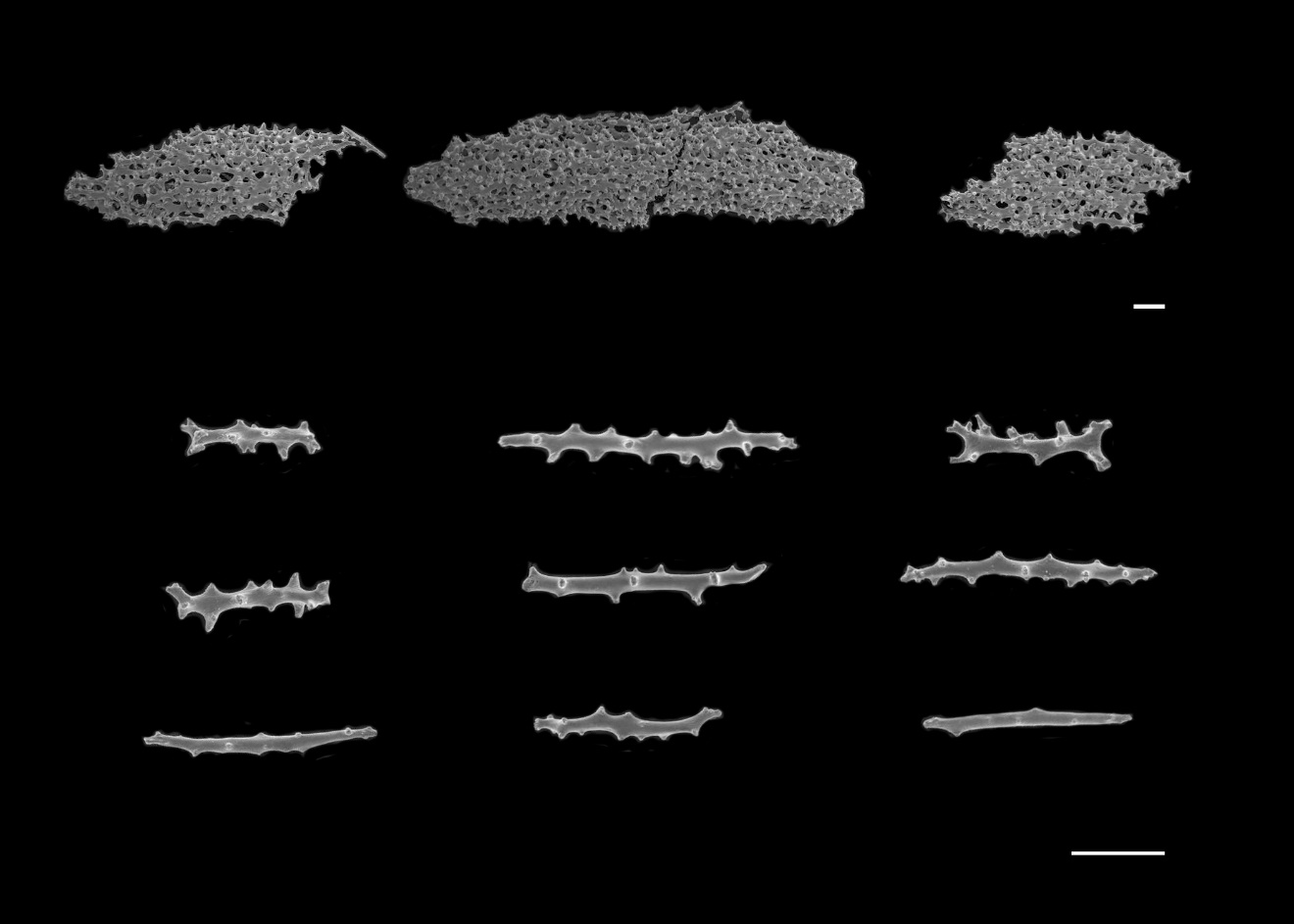


Plate of representative sclerites of specimen CLP2_A03. Scale bars represent 100 µm.

**Scripts and commands**

Trimmomatic script:

#1/bin/bash

for dir in $@

do

cat $dir/*R1*.fastq > $dir/combined-1.fastq

cat $dir/*R2*.fastq > $dir/combined-2.fastq

FORWARD=$dir/combined-1.fastq;

REVERSE=$dir/combined-2.fastq;

FORWARD=$(echo $FORWARD);

REVERSE=$(echo $REVERSE);

nohup trimmomatic PE -threads 8 $FORWARD $REVERSE $dir/paired-1.fastq.gz $dir/unpaired-1.fastq.gz $dir/paired-2.fastq.gz $dir/unpaired-2.fastq.gz ILLUMINACLIP:/opt/Trimmomatic-0.32/adapters/NexteraPE-PE.fa:2:30:10 LEADING:3 TRAILING:3 SLIDINGWINDOW:4:15 MINLEN:3$

done

Spades script:

for dir in $@

do

cd $dir

FORWARD=paired-1.fastq.gz

REVERSE=paired-2.fastq.gz

UNPAIRED1=unpaired-1.fastq.gz

UNPAIRED2=unpaired-2.fastq.gz

nohup spades.py --pe1-1 $FORWARD --pe1-2 $REVERSE --pe1-s $UNPAIRED1 --pe1-s $UNPAIRED2 -t 16 -o spades_assembly &

cd ../

done

BBDuk: commands:

[ktrim=r, k=27, hdist=1, edist=0, ref=nextera.fa.gz, qtrim=rl, trimq=6, minlength=10, ordered=t, qin=33, in=input1.fastq, in2=input2.fastq, out=output1.fastq, out2=output2.fastq]

**Acknowledgements**

This publication was made possible by the National Oceanic and Atmospheric Administration, Office of Education Educational Partnership Program award NA16SEC4810009. Its contents are solely the responsibility of the award recipient and do not necessarily represent the official views of the U.S. Department of Commerce, National Oceanic and Atmospheric Administration. Research cruise funding provided by Texas Parks and Wildlife Department—Artificial Reef Program (Grant No. 475342, 2016–2018) and research expenses supported by award NA16SEC4810009 and New Hampshire-INBRE through an Institutional Development Award (IDeA), P20GM103506, from the National Institute of General Medical Sciences of the NIH. Sequencing costs supported by an Institutional Grant (NA14OAR4170102) to the Texas Sea Grant College Program from the National Sea Grant Office, National Oceanic and Atmospheric Administration, U.S. Department of Commerce. Funds for contracted labor for SEM preparation and imaging were provided by Southeast Deep Coral Initiative to CSS, Inc, led by NOAA National Centers for Coastal Ocean Science (NCCOS) with support from NOAA Deep Sea Coral Research and Technology Program. We would like to thank Andrew Schuler and Peter Etnoyer of the NOAA Deep Sea Coral Research and Technology Program for their support for the SEM analyses and Steve Morton of the NCCOS Marine Biotoxins Programs for allowing us use of the SEM.

**Literature cited:**

Bankevich A, Nurk S, Antipov D, Gurevich AA, Dvorkin M, Kulikov AS, Lesin VM, Nikolenko SI, Pham S, Prjibelski AD. 2012. SPAdes: a new genome assembly algorithm and its applications to single-cell sequencing. J Comput Biol. 19(5):455-477.

Bayer FM. 1961. The shallow-water Octocorallia of the West Indian region. Studies on the Fauna of Curaçao and other Caribbean Islands. 12(1):1-373.

Bolger AM, Lohse M, Usadel B. 2014. Trimmomatic: a flexible trimmer for Illumina sequence data. Bioinformatics. 30(15):2114-2120.

Curley SJ. 2011. The ship that would not die: USS Queens, SS Excambion, and USTS Texas Clipper. Texas A&M University Press. pp.117.

Edgar RC. 2004. MUSCLE: multiple sequence alignment with high accuracy and high throughput. Nucleic Acids Res. 32(5):1792-1797.

Lanfear R, Calcott B, Ho SY, Guindon S. 2012. PartitionFinder: combined selection of partitioning schemes and substitution models for phylogenetic analyses. Molecular biology and evolution. 29(6):1695-1701.

Lanfear R, Frandsen PB, Wright AM, Senfeld T, Calcott B. 2017. PartitionFinder 2: new methods for selecting partitioned models of evolution for molecular and morphological phylogenetic analyses. Molecular biology and evolution. 34(3):772-773.

Stamatakis A. 2014. RAxML version 8: a tool for phylogenetic analysis and post-analysis of large phylogenies. Bioinformatics. 30(9):1312-1313.
